# Laparoscopic resection is not superior to endoscopic resection for relative small gastric gastrointestinal stromal tumors: A propensity score-matched study

**DOI:** 10.3389/fonc.2022.1015916

**Published:** 2022-10-13

**Authors:** De-Liang Li, Yang-Yang Zhou, Ji-Yu Zhang, Dan Liu, Li-Xia Zhao, Bing-Rong Liu

**Affiliations:** Department of Gastroenterology and Hepatology, The First Affiliated Hospital of Zhengzhou University, Zhengzhou, China

**Keywords:** endoscopic resection, laparoscopic resection, gastric GISTs, tumors, comparison

## Abstract

**Background and Aim:**

Endoscopic resection (ER) and laparoscopic resection (LAP) have been recommended for the treatment of gastric gastrointestinal stromal tumors (GISTs) less than 2 cm. However, the therapeutic approach for gastric GISTs between 2 and 5 cm in diameter is still under debate. In this retrospective study, we aimed to evaluate the feasibility, efficacy, and safety of ER for gastric GISTs (2–5 cm) compared with LAP.

**Methods:**

From January, 2011 to January, 2018, 197 patients with GISTs at our institution with tumor diameter between 2 and 5 cm were included in our study. Clinical baseline characteristics, histopathological results, and perioperative outcomes were collected and compared in all the patients. Propensity score matching (PSM) methods were used to balance baseline characteristics.

**Results:**

There was no significant difference in age (p = 0.246), gender (p = 0.572), tumor location (p = 0.333), pathological risk classification (p = 0.543), Ki-67 index (p = 0.212), and follow-up time (p = 0.831) in the ER and LAP groups. However, significance difference was found in times to liquid diet intake (4.45 ± 1.2 vs. 5.40 ± 1.5 days, p = 0.013) and hospital stays (7.72 ± 1.1 vs. 10.01 ± 1.3 days, p < 0.001). During the follow-up period, there was one recurrence in the ER group vs. two recurrences in the LAP group. After PSM, the tumor size was balanced between the two groups with 49 patients in each group. The times to liquid diet intake (4.18 ± 1.3 vs. 5.16 ± 1.6 days, p = 0.042) and hospital stay days (7.12 ± 1.1 vs. 9.94 ± 1.3, p < 0.0001) were still short in the ER group.

**Conclusions:**

ER is more associated with a quick postoperative recovery than LAP. ER could be an alternative approach for gastric GISTs (2–5 cm). However, the long-term follow-up outcomes are still unclear and random control trials are needed.

## Introduction

Gastrointestinal stromal tumors (GISTs) are the most common mesenchymal lesions, accounting for 1% of gastrointestinal (GI) tumors (1). It can present throughout the GI tract, predominately in the stomach and small intestine (2–4). The clinical incidence of GISTs is about 1 per 100,000 annually with 54% men and 46% women (1). GISTs show a wide variety of clinical manifestations due to potential malignancy, the prognosis of which is difficult to predict and related to tumor size, mitotic index, KI-67, proliferative index, etc. (5).

With the development of the endoscopic technique, the detective rate of GISTs obviously increased (6). For small GISTs (<2 cm), endoscopic surveillance and follow-up are recommended. For non-metastatic GISTs with size >2 cm, tumor resection is the first-line treatment according to the National Comprehensive Cancer Network (NCCN) guidelines. GISTs have a low rate of lymphatic metastasis; local resection without lymphadenectomy is admitted. Large GISTs (>5 cm) appear to be more aggressive in morphologic features and proliferation activity, so laparoscopic resection (LAP) or surgery is more beneficial for complete resection. However, it is still controversial for the treatment criteria of relatively small GISTs (2–5 cm). Recently, the safety and effectivity of endoscopic resection (ER) have been proven in many studies (7, 8). Compared to LAP, ER is more minimally invasive, although in some cases has limited procedure space. Only a few reports have referred the outcome of ER for gastric GIST (2–5 cm) (9), and little is known about the clinical outcomes of ER procedures in comparison with LAP for gastric GISTs (2–5 cm). Therefore, this retrospective study aimed to compare the safety and feasibility of ER and LAP for gastric GISTs 2–5 cm in diameter in our center.

## Materials and methods

### Patients

This retrospective study was conducted in the first affiliated hospital of Zhengzhou University from January, 2011 to January, 2018. Patients who underwent curative resection and were confirmed with gastric GISTs by postoperative histopathological examinations were included in our study. Among these patients, 197 patients with tumor between 2 and 5 cm in diameter treated by ER were randomly matched (1:1) using propensity score matching (PSM) methods to patients who underwent LAP. The match criteria were as follows: 1) average age difference between groups <5 years; 2) gender consistency; 3) mean tumor maximum diameter difference between groups <0.5 cm; and 4) consistency of whether neoadjuvant imatinib was administered.

Baseline characteristics and clinicopathological data were obtained from the electronic medical database of our hospital, including age, sex, clinical symptoms, tumor locations, tumor size, and histopathological results. Operation-related outcomes were also collected, including operative duration, intraoperative and postoperative adverse events, time to oral intake, and length of postoperative stay. The median follow-up period, recurrence rate, and overall survival time were also recorded and compared. Informed consents were signed from all the inclusion patients after explaining each treatment management and the possible risks. This study was approved by the Institutional Review Board of The First Affiliated Hospital of Zhengzhou University.

### Surgical procedures

Procedures were performed under anesthesia with tracheal intubation. Endoscopic resection includes endoscopic submucosal dissection (ESD) and endoscopic full-thickness resection (EFTR). The ESD procedure mainly included three steps as follows: (i) identifying and marking the tumor boundaries; (ii) injecting the saline water mixed with indigo carmine to the submucosal layer; and (iii) incising the mucosal layer with a hook knife and dissecting the tumor using the IT knife.

EFTR enables the resection of tumors that originated from the muscularis propria or serosal layer. The technique mainly has five steps: (i) marking the dots around the tumor using argon plasma coagulation; (ii) injecting the saline water into the submucosal layer; (iii) incising and dissecting the mucosa; (iv) resecting on full thickness; and (v) clipping the incision.

The LAP procedure includes the following steps: (i) Establish an initial access site and use carbon dioxide to create the pneumoperitoneum at a pressure of 12 mmHg. (ii) Four additional trocars are placed in an inverted trapezoidal setting in both procedures. (iii) Before resection, check out the overall abdominal situation. (iv) The wedge resection or gastrectomy is performed according to the tumor size and location. (v) Make sure that the gastric-wall defect is closed with a laparoscopic linear stapler or hand-sewn.

### Histopathological evaluation

The resected specimens were fixed and sent to the pathological department. Hematoxylin and eosin (HE) and immunohistochemicals were applied in all specimens, including the cell type, the mitotic index (MI), and the risk and related immunohistochemical markers.

### Follow-up strategy

All patients received telephone follow-up or outpatient services within 1 month after being discharged to determine whether short-term complications occurred. Regular outpatient follow-up was performed according to the following strategy: 3, 6, and 12 months and yearly thereafter. Endoscopy and computed tomography (CT) were performed on every outpatient follow-up. Endoscopic examination was performed to assess the healing of the wound, and computed tomography (CT) evaluated the extent of the tumor. Recurrence was confirmed by CT scans and endoscopic ultrasound (EUS) was applied if necessary.

### Statistical analysis

The main observational indicators included patient clinicopathological characters, perioperative results, and follow-up outcomes. Data were analyzed using SPSS 22.0 (International Business Machines Corporation, Chicago, IL, USA). Continuous data are shown as mean and standard deviation. Categorical data are expressed as frequency. Statistical differences between groups were performed using Student’s *t*-test for continuous data and chi-square test or Fisher’s exact test for categorical data. p < 0.05 was considered statistically significant.

## Results

### Clinical baseline data

The patients with GISTs who underwent surgical treatment in our hospital were retrospectively analyzed. According to the inclusion and exclusion criteria, 197 cases were finally included in this study with 74 cases in the ER group and 123 cases in the LAP group. The average age of the patients in the ER group was 59.5 years, and the ratio of male to female patients was 30:44; the average age of the patients in the laparoscopic group was 57.6 years, and the ratio of male to female patients was 57:66. The most common location distribution of tumors was the gastric fundus in both groups. There was no significant difference in age (59.50 vs. 57.57, p = 0.246), gender (p = 0.572), and tumor location (p = 0.333) between the two groups, but there was a statistically significant difference in tumor size (27.59 mm vs. 33.24 mm, p < 0.001).

### Operative and pathological outcomes

All the 197 patients underwent tumor resection successfully. The ER group had significant advantages in postoperative fasting time and postoperative hospital stay, which were significantly shorter than those in the laparoscopic group (4.45 ± 1.2 days vs. 5.40 ± 1.5 days, p = 0.013; 7.72 ± 1.1 days vs. 10.01 ± 1.3, p < 0.001). There was no significant difference in operation time (98.80 ± 12.0 min vs. 110.79 ± 15.5 min, p = 0.110).

According to histological outcomes, 11, 26, 10, and 11 patients were identified to have very low-, low-, middle-, and high-risk GISTs in the ER group, respectively. In the LAP group, 16, 54, 37, and 16 patients were identified to have very low-, low-, middle-, and high-risk GISTs, respectively. The risk classifications had no difference between the two groups (p = 0.543). Moreover, 33, 29, and 12 patients were identified to have a Ki-67 index of <5%, 5%–10%, and >10% in the ER group, respectively. In the LAP group, 39, 63, and 21 patients were identified to have a Ki-67 index of <5%, 5%–10%, and >10%, respectively. The Ki-67 index had no significant difference between the two groups (p = 0.212).

### Postoperative compilations

Six complications in 74 cases were found in the ER group, namely five cases of perforation and one case of pleural effusion. Eight complications in 123 cases in the LAP group were found, namely three cases of bleeding, two cases of intestinal obstruction, one case of perforation, one case of incision fat liquefaction, and one case of unstable vital signs. The postoperative complication rate had no difference between the two groups (p = 0.853).

In the ER group, two cases were converted to laparoscopic treatment during the operation, and 10 cases in the laparoscopic group were converted to open surgery due to the inability to resect the tumor entirely (**Table 2**).

### Follow-up

A total of 197 patients were followed up by gastroscopy and CT scans. The mean follow-up times were 54.27 ± 12.6 and 55.96 ± 13.5 months, respectively. Ten patients in the LAP group were treated by imatinib, but only one patient received imatinib therapy. During the follow-up period, there was one recurrence in the ER group and two recurrences in the LAP group.

### Propensity score matching analysis

Because the difference in tumor size between the two groups was obvious, it was considered a confounder. Propensity score matching (PSM) (1:1) was performed on the two groups of cases, and 49 pairs were successfully matched. A total of 98 matched patients were statistically analyzed, and there were no significant differences in age (58.82 vs. 57.31, p = 0.462), gender (p = 0.682), tumor location, (p = 0.727), and tumor size (29.78 mm vs. 29.53 mm, p = 0.897) between the two groups (**Table 1**).

**Table 1:** Baseline characteristics of patients with gastric GISTs (2–5 cm) undergoing ER or LAP in the cohort.

Variable	Before matching			After matching		
	ER (n=74)	LAP (n=123)	P	ER (n=49)	LAP (n=49)	P
Age average±SD, year	59.50±13.45	57.57±12.15	0.246	58.82±14.13	57.31±16.13	0.462
Gender			00.572			0.682
Male	30	57		29	31	
Female	44	66		20	18	
Tumor size mean mm	27.59	33.24	<0.001	29.78	29.53	0.897
Location			0.333			0.727
Cardia	2	4		1	1	
Gastric fundus	48	73		34	31	
Gastric body	23	41		14	16	
Gastric antrum	1	5		0	1	
Risk classification			0.543			
Very low	11	16				
Low	26	54				
Middle	10	37				
High	11	16				
Ki-67			0.212			
<5	33	39				
05-10	29	63				
>10	12	21				
Follow-up			0.831			
Time average±SD, month	54.27±12.6	55.96±13.5		51.55 (30-98)	57.90 (33-93)	
Recurrence	1	2		1	1	

SD, standard deviation; ER, endoscopic resection; LAP, laparoscopic resection.

There were two complications in 49 cases in the ER group, namely two cases of perforation. There were two complications in 49 cases in the laparoscopic group, namely one case of bleeding and one case of perforation. In the ER group, two cases were converted to laparoscopic treatment during the operation, and three cases in the LAP group were converted to open surgery due to inability to resect the tumor entirely (**Table 2**). The postoperative complications had no difference between the two groups (p = 1.00). However, the postoperative hospital stay (p < 0.01) and the time to first oral intake (p < 0.001) were still shorter in the ER group. Moreover, one patient experienced recurrence in each group (p = 1.00).

**Table 2:** Therapeutic outcomes of included patients.

Variable	Before matching			After matching			
	ER (n = 74)	LAP (n = 123)	P	ER (n = 49)		LAP (n = 49)		P	
Operation time average ± SD, min	98.80 ± 12.0	110.79 ± 15.5	0.11	94.55 (39-211)		105.9 (30-250)			0.235
Transfer operation approach	2	10		2		3			
Time to liquid diet average ± SD, days	4.45 ± 1.2	5.40 ± 1.5	0.013	4.18 ± 1.3		5.16 ± 1.6			0.042
Hospital stay average ± SD, days	7.72 ± 1.1	10.01 ± 1.3	<0.001	7.12 ± 1.1		9.94 ± 1.3			<0.001
Adverse events			0.853						0.001
Bleeding	0	3		0		1			
Perforation	5	1		2		1			
Pleural effusion	1	1		0		0			
Intestinal obstruction	0	2		0		0			
Others	0	1		0		0			
Postoperative imatinib therapy	1	10		1		5			

## Discussion

Gastrointestinal stromal tumors (GISTs), the most common mesenchymal neoplasms, have been shown to be derived from interstitial Cajal cells (1). With a wide variety of clinical manifestations, it can be present throughout the GI tract, predominantly in the stomach (50%–60%). The incidence of GISTs is about 1 per 100,000 annually, which is still a threat to human health due to the potential malignance existing in all the GISTs.

Complete resection is still the first-line treatment for non-metastasis GISTs (10). Most GISTs rarely have lymphatic metastasis, so local resection without lymphadenectomy is admitted. Endoscopic surveillance and follow-up are currently recommended for GISTs larger than 2 cm. Sometimes it burdens patients emotionally and financially, so tumor resection is often selected. Large GISTs (>5 cm) appear to be more aggressive in morphologic features and proliferation activity, so laparoscopic resection (LAP) or even more surgery is more beneficial for complete resection. However, it is still controversial about the treatment criteria for relatively small GISTs (2–5 cm). With the development of laparoscopic and endoscopic techniques, these minimally invasive operations can achieve both surgical negative margin and an intact capsule of the GISTs. Few studies have made a comparison of the safety and effectiveness between laparoscopic resection and endoscopic resection. In this study, we thoroughly examined two types of minimally invasive resection treatment for gastric GISTs with diameters ranging from 2 to 5 cm.

Through previous review of literature, guidelines, and a summary of clinical experience, we determined that the tumor diameter is an important factor affecting the selection of approach (11). It has certain effects on operation time, postoperative recovery, and even disease prognosis. By comparing the clinical baseline data of the two groups, it was found that the tumor size in the endoscopic group was significantly lower than that in the laparoscopic group, so we included “tumor diameter” into the PSM variable for analysis. The location of the tumor also affects the choice of surgery to some extent (11). In the laparoscopic group, 10 cases were converted to open surgery, of which five cases were located in the lesser curvature of the gastric body (near the posterior wall of the gastric angle), four cases were located in the greater curvature of the fundus, and one case was located in the greater curvature of the gastric body. We therefore included “tumor location” in the analysis.

Considering that too many covariates will result in too much loss of the output data of this study, we only included patient age and gender in the variable analysis. In the end, 49 cases in the endoscopic treatment group and 49 cases in the laparoscopic treatment group were successfully matched. After matching, there were no significant differences in patient age, gender, tumor size, and tumor diameter between the two groups. In terms of operation-related indicators, the postoperative fasting time and postoperative hospital stay of the endoscopic group were significantly shorter than those of the laparoscopic group, which showed the minimally invasive advantages of endoscopic treatment, consistent with the results of Wang et al. In terms of operation time, the advantage of the endoscopic group is not obvious, and there is no statistical difference between the two groups. Considering that the data range included in this study is between, 2011 and, 2018, it represents the continuous development of domestic endoscopic technology on the basis of ESD. In the past 10 years, the performance of operations, endoscopic care cooperation, and suturing technology has been unstable, and it has not reached the maturity of laparoscopic technology in the same period; these cases were not operated on by the same endoscopist.

In terms of complications, no difference was observed between the two groups. However, Pang et al. compared endoscopic (n = 268) and surgical (including open and laparoscopic resection, n = 141) resection of small GISTs (<5 cm) in, 2019 (8). They discovered that endoscopic resection was always associated with a lower incidence of complications even after a propensity score matching analysis (3.6% vs. 13.1%, p = 0.026), suggesting endoscopic resection as a safe and promising alternative treatment for gastric GIST. Other previous studies in the past decades found that endoscopy had a similar or higher incidence of complications compared with laparoscopy; the conclusion was consistent with ours (12, 13). Therefore, whether ER has the advantage of safety should be verified in future studies, but the advantage of promoting quick recovery has no doubt in our study cohort.

The majority of the lesions may be endoscopically removed with a full capsule when seen macroscopically. The burnt edge on the capsule, however, may lead to a pathological diagnosis of R1 resection when examined under a microscope. Even in the hands of seasoned professionals, this is sometimes unavoidable and is not seen as learning-curve-related. Fortunately, research has shown that patients who receive R0 and R1 resection do not significantly vary in terms of relapse-free survival (RFS) (14, 15). Thus, despite resection margin involvement, endoscopic resection for stomach GISTs was still seen as a viable option (16), and the margin status was not analyzed in our study.

During the follow-up period, one patient died due to recurrence and metastasis in the endoscopic group but none in the laparoscopic group. There was one case of recurrence and metastasis in the endoscopic group and two cases of recurrence and metastasis in the laparoscopic group. The pathological results of these three patients suggested that they were all high-risk types. The recurrence rate was similar between the two groups. However, one study did find a high local recurrence rate (5.8%) after endoscopic enucleation of 86 GISTs, especially in large tumors (17), though complete endoscopic resection was achieved in all cases in this study. Our results also showed that patients in the ER group had smaller tumors than those in the surgical group. It may have been expected that endoscopic resection should also have advantages in OS and RFS than in surgical resection, but this was not the case. This may be due to the small sample size and short follow-up period. Long-term outcomes of GISTs still require confirmation in more prospective studies or multicenter studies.

This study has some limitations. It is a single-center retrospective study with a relatively small sample size, and there is a certain selection bias in the sample data. Therefore, it is necessary to further verify the above results through a multicenter prospective study. Endoscopic treatment is becoming more and more mature, and data over a longer time span could be collected for comprehensive analysis. The evaluation of patients’ satisfaction with perioperative management was not included in this study, and it is recommended to add this indicator in future research.

## Conclusion

In conclusion, in this retrospective study, the clinical baseline data of the two groups of patients were unevenly distributed. After propensity score matching, there was no statistical difference in age, gender, tumor size, and tumor diameter between the two groups. The matched endoscopic group was significantly better than the laparoscopic group in terms of postoperative fasting time and postoperative hospital stay. During the average follow-up of 55.12 months, there was no significant difference in the postoperative long-term efficacy between the two groups. Endoscopic resection treatment is safe and feasible for primary gastric stromal tumors with a diameter of 2–5 cm, and further research needs to be confirmed by multicenter prospective trials.

## Data availability statement

The original contributions presented in the study are included in the article/supplementary material. Further inquiries can be directed to the corresponding author.

## Ethics statement

This study was reviewed and approved by The First Affiliated Hospital of Zhengzhou University. The patients/participants provided their written informed consent to participate in this study.

## Author contributions

Study concept and design: D-LL, B-RL. Manuscript writing: D-LL, Y-YZ. Technical and material support: Y-YZ, J-YZ, DL, L-XZ. Data collection: Y-YZ, J-YZ, DL, L-XZ. Revision of manuscript: B-RL. All authors contributed to the article and approved the submitted version.

## Funding

1. Zhongyuan talent program (NO: ZYYCYU202012113) 2. The key R&D program of Henan Province (No., 222102310038) 3. Henan key medical laboratory: Innovative technology for minimally invasive treatment of digestive endoscope.

## Conflict of interest

The authors declare that the research was conducted in the absence of any commercial or financial relationships that could be construed as a potential conflict of interest.

## Publisher’s note

All claims expressed in this article are solely those of the authors and do not necessarily represent those of their affiliated organizations, or those of the publisher, the editors and the reviewers. Any product that may be evaluated in this article, or claim that may be made by its manufacturer, is not guaranteed or endorsed by the publisher.
